# Late-onset midgut volvulus 8 years following a neonatal omphalocele repair: a case report and review

**DOI:** 10.1093/jscr/rjaf604

**Published:** 2025-08-11

**Authors:** Hadjar Nassiri, Loubna Aqqaoui, Houda Oubejja, Fouad Ettayebi

**Affiliations:** Université Mohammed V de Rabat, Faculté de medecine et de pharmacie de Rabat, BP 6203 – Rabat‑Instituts, Avenue Mohammed Belarbi El Alaoui, 10100 Rabat, Morocco; Ibn Sina University Hospital Center, Children's Hospital, Avenue Ibn Rochd, Quartier Souissi, 10100 Rabat, Morocco; Abdelmalek Essaadi University, Laboratory of Life and Health Sciences, Faculty of Medicine and Pharmacy of Tangier, B.P. 365 Gzenaya, KM 15, Route de Rabat – 90080 Tanger, Morocco; Université Mohammed V de Rabat, Faculté de medecine et de pharmacie de Rabat, BP 6203 – Rabat‑Instituts, Avenue Mohammed Belarbi El Alaoui, 10100 Rabat, Morocco; Ibn Sina University Hospital Center, Children's Hospital, Avenue Ibn Rochd, Quartier Souissi, 10100 Rabat, Morocco; Université Mohammed V de Rabat, Faculté de medecine et de pharmacie de Rabat, BP 6203 – Rabat‑Instituts, Avenue Mohammed Belarbi El Alaoui, 10100 Rabat, Morocco; Ibn Sina University Hospital Center, Children's Hospital, Avenue Ibn Rochd, Quartier Souissi, 10100 Rabat, Morocco; Université Mohammed V de Rabat, Faculté de medecine et de pharmacie de Rabat, BP 6203 – Rabat‑Instituts, Avenue Mohammed Belarbi El Alaoui, 10100 Rabat, Morocco; Ibn Sina University Hospital Center, Children's Hospital, Avenue Ibn Rochd, Quartier Souissi, 10100 Rabat, Morocco

**Keywords:** omphalocele, midgut volvulus, Ladd’s procedure, late-onset complication, case report

## Abstract

Omphalocele is a congenital abdominal wall defect. It is frequently associated to other anomalies such us intestinal malrotation, though this is frequently underdiagnosed. We report a rare case of an 8-year-old boy with a history of neonatal surgery for a small type I omphalocele. The child was asymptomatic since his neonatal primary repair. Presented acutely with signs of intestinal obstruction. Imaging suggested high-grade obstruction, and emergency laparotomy revealed a midgut volvulus caused by tight Ladd’s bands around a narrowed mesenteric base. A Ladd’s procedure was performed, including detorsion, band division, bowel repositioning, and prophylactic appendectomy. The patient recovered uneventfully. This case highlights the potential for late-onset volvulus in patients with prior omphalocele repair, underlining the importance of long-term follow-up and a high index of suspicion in acute presentations. It also raises considerations regarding the prophylactic role of Ladd’s procedure during initial repair, especially in settings equipped for ongoing surveillance.

## Introduction

An omphalocele is a congenital defect of the abdominal wall, with protrusion of the abdominal contents through the umbilical ring. It is covered by a membranous sac [1]. Its estimated incidence is 1/4000 to 1/5000 live births [2].

During the 6th week of gestation, the midgut undergoes a physiologic herniation through the umbilicus, allowing a counterclockwise rotation around the superior mesenteric artery and intestinal elongation. By 10 to 12 weeks the midgut undergoes reduction into the abdominal cavity and returns to its normal anatomical position. Omphalocele represents a failure of this process, though exactly how this develops is unclear [3].

It can measure between 2 and 10 cm and 40%–80% of all cases will have at least one concurrent anomaly, including cardiac defects, chromosomal abnormalities, and gastrointestinal malformations such as intestinal malrotation [4]. In fact, infants with an omphalocele typically have the intestine in a non-rotated position, that is with the small bowel loops in the right abdominal quadrants and the large bowel loops in the left quadrants [5].

Current practice, in regard to prevention of midgut volvulus in patients with abdominal wall defects, is variable [6]. A Ladd’s procedure is not routinely performed in the majority of these patients as it is thought that post repair adhesions are enough to provide bowel fixation and prevent midgut volvulus [6].

While most of volvulus occur earlier in the neonatal period, late presentations in older children yet rare but possible. Especially if the malrotation is not explored.

## Case report

An 8-year-old male child, with a history of neonatal surgery for Type 1 omphalocele (small defect, containing bowel only), presented to the emergency department with acute onset of abdominal pain, bilious vomiting, and marked abdominal distension, making a frank acute occlusive syndrome. The child had no prior history of similar episodes and had been in good health since the neonatal surgery, which involved primary closure of the abdominal defect.

On clinical examination, the patient appeared pale and lethargic. Vital signs indicated tachycardia and mild hypotension. The abdomen was grossly distended, with generalized tenderness and guarding, a 1 cm scar around the navel. Suggestive of bowel sounds were absent.

**Figure 1:**
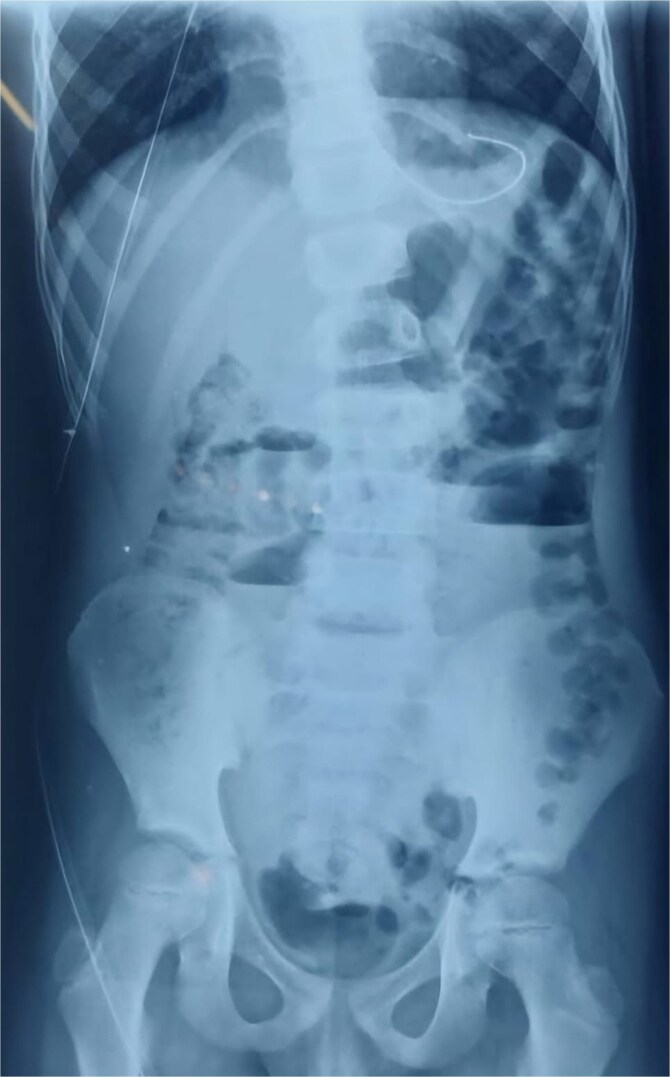
X-ray image at admission showing multiple dilated bowel loops with air-fluid levels.

**Figure 2:**
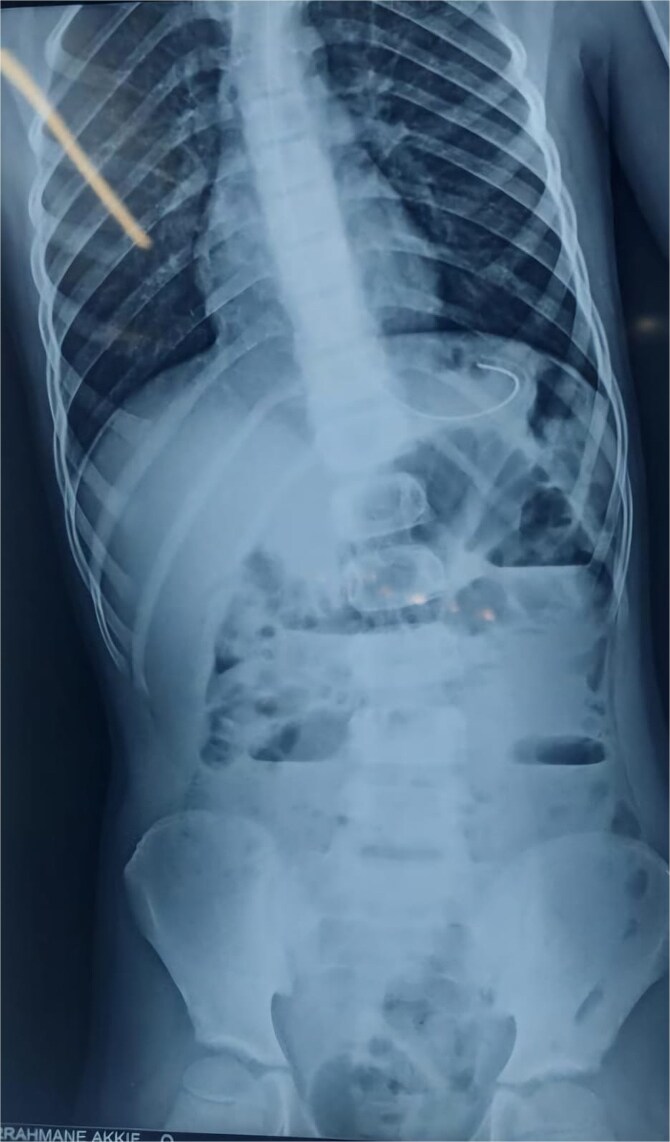
X-ray image after 6 h of admission, showing persistence of air-fluid levels.

An abdominal X-ray showed multiple dilated bowel loops with air-fluid levels (Figs 1 and 2), consistent with a high-grade intestinal obstruction. Given the patient’s surgical history, a diagnosis of volvulus secondary to intestinal malrotation was strongly considered.

**Figure 3:**
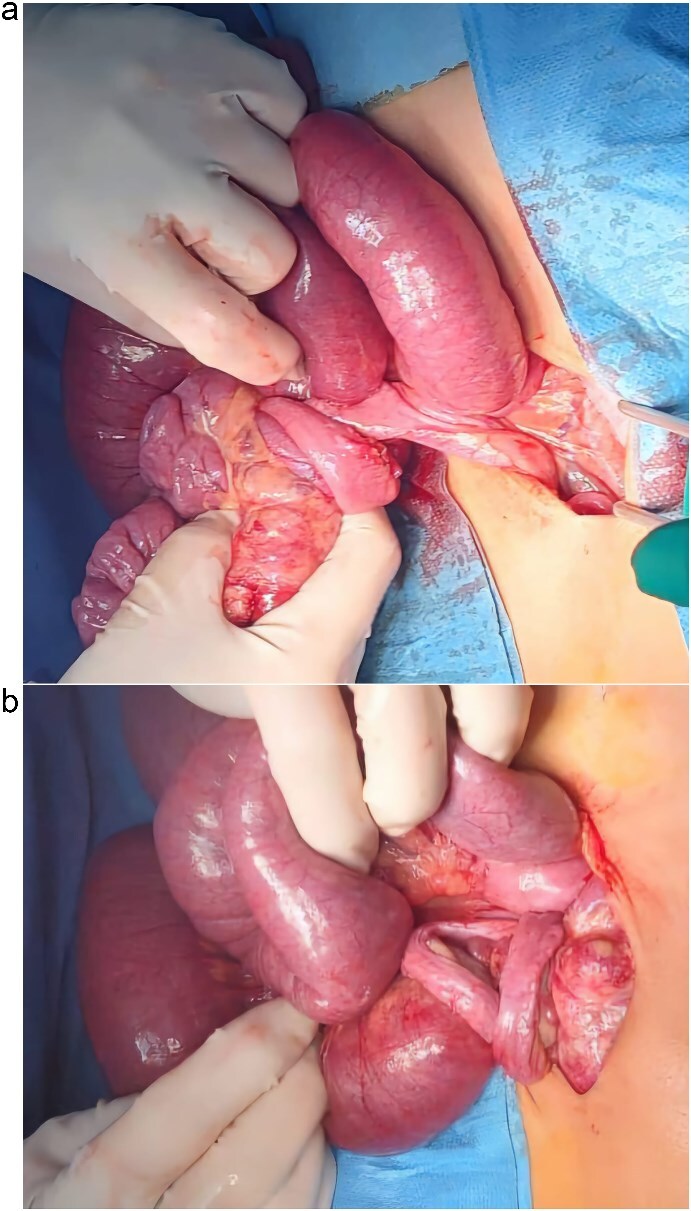
(a and b) Preoperative images showing the midgut volvulus found in the patient after exploration.

**Figure 4:**
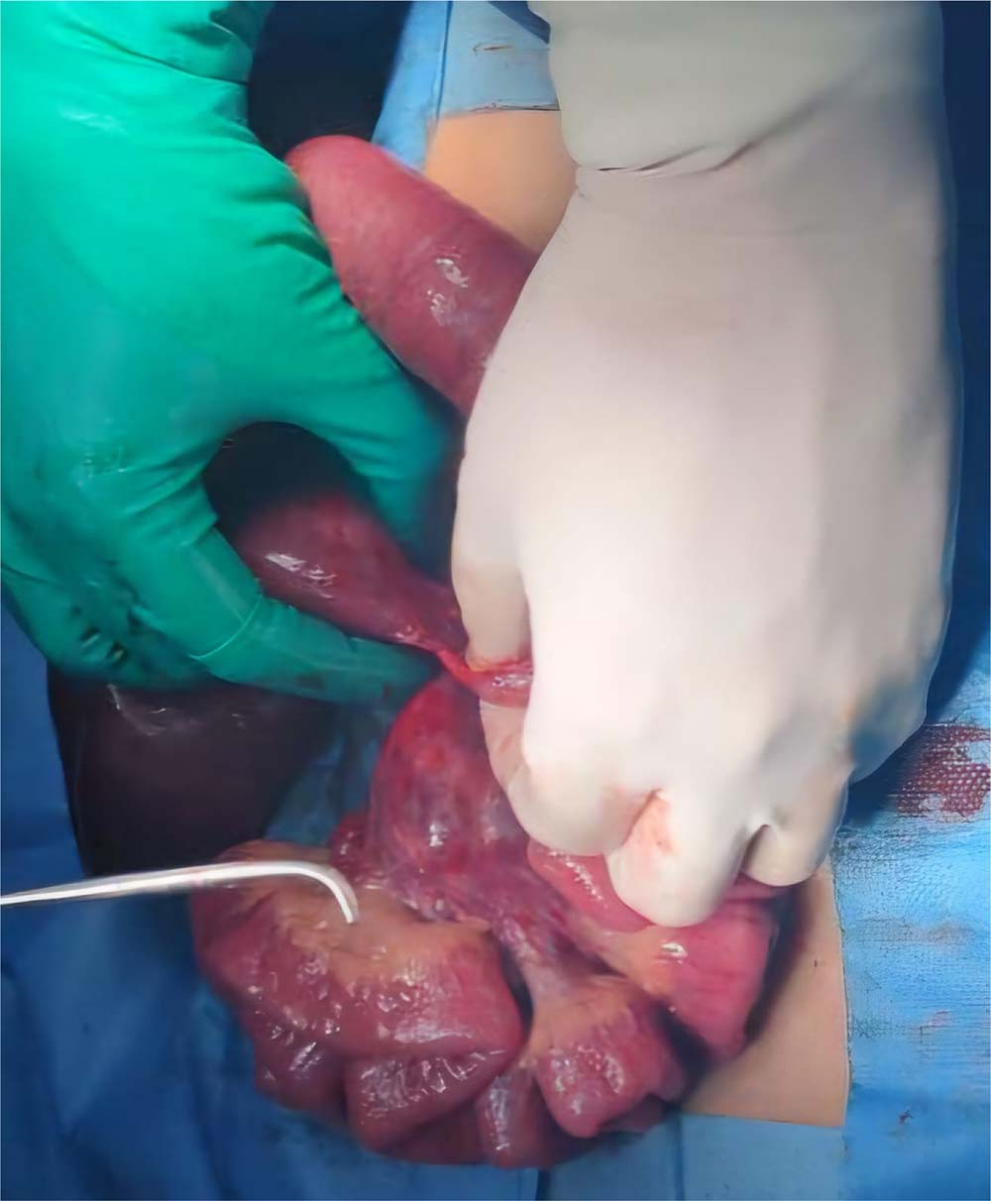
Preoperative image showing a Ladd band.

**Figure 5:**
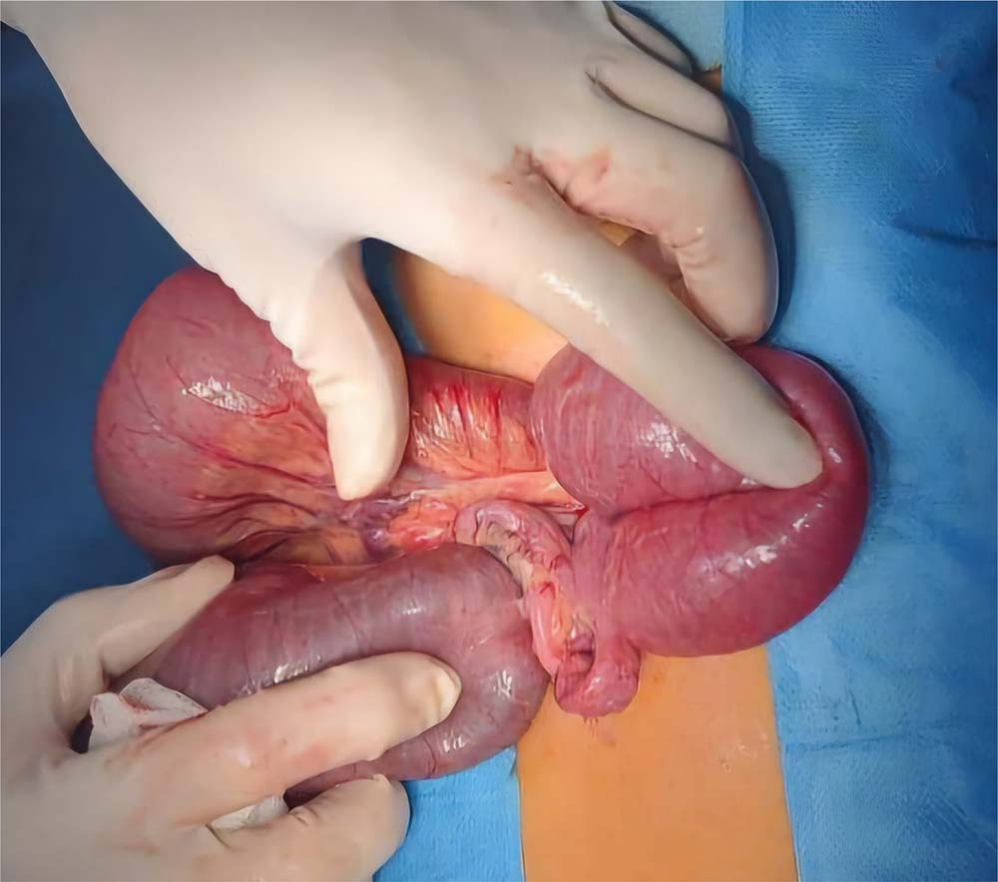
A preoperative picture demonstrating the placement of the bowel in a non-rotated position.

The patient was admitted for emergency exploratory laparotomy. Intraoperatively, a midgut volvulus was identified (Fig. 3a and b), caused by tight Ladd’s bands (Figs. 4) leading to twisting of the small bowel around a narrowed mesenteric base. There were no signs of bowel necrosis. A Ladd procedure was performed, which included detorsion of the volvulus, division of Ladd’s bands, and placement of the bowel in a non-rotated position to prevent recurrence (Fig. 5). The appendix was also removed prophylactically.

Postoperatively, the child recovered uneventfully, resumed oral feeding on postoperative Day 3, and was discharged home on Day 7 in good condition. He presented no complication during his stay at our department nor at follow ups.

## Discussion

Omphalocele is a congenital anomaly that results from the none return of intestines to the abdominal cavity after normal physiological herniation into the umbilical cord at Weeks 6–10 of gestation [7]. However, this interruption often results in abnormalities in intestinal rotation and fixation, leading to malrotation. That can result in mortality and fatal morbidities [8].

Malrotations in neonates born with omphalocele has a low incidence, this is probably because they are underreported. Its occurrence is not related to the size of the abdominal wall defect [9]. Lauriti *et al*. also noted that Ladd's procedure—a surgical intervention often used to manage malrotation—did not decrease the risk of postoperative volvulus for these neonates [9]. This finding defies the long-held belief that Ladd's procedure is indeed prophylactic for this population of patients.

In our department, we do not perform a Ladd’s procedure while treating an omphalocele. Given the fact that, we are a tertiary care unit, involved in the management of complex congenital pathologies not only in our region but also in the whole country. This practically means that complicated patients, with volvulus for example would return to our center for emergency care. This allows for longitudinal follow-up and surveillance.

Incidence of associated malformations in omphalocele patients is reported to be between 27% and 63% from various studies, revealing the high rate of coexisting anomalies [7]. More recently, there has been growing recognition of non-classical intestinal anomalies in patients with small omphaloceles, suggesting that the spectrum of intestinal involvement is much broader than previously believed [10].

Although such complications are rarely described in the literature, incidence in patients with omphalocele is reported to be higher than in the general population [7]. Specifically, incidence of malrotation is reported at 10% in patients with omphalocele, while presence of midgut volvulus is observed in 3.3% of cases [7]. On the other hand, the incidence of midgut volvulus in the general population is estimated to be 1.7 to 60 per 100 000 [7].

This has implications for surgical decision making. While exploration of the mesenteric base during initial repair in a small omphalocele is a relatively simple matter, it might still slightly increase the risk of postoperative complications such as ileus or adhesions [11, 12].

## Conclusion

This case highlights a rare but serious complication of undiagnosed intestinal malrotation following omphalocele repair. Clinicians should maintain a high index of suspicion for volvulus in any child with a history of omphalocele presenting with acute abdominal symptoms. This case also emphasizes the importance of long-term follow-up in children with congenital abdominal wall defects, as complications can arise beyond the immediate postoperative period.
